# Integrative Mendelian Randomization and Multiomics Analysis Identifies CAF‐Derived Complement Factor B as a Prognostic Biomarker and Therapeutic Vulnerability in Breast Cancer

**DOI:** 10.1155/humu/2179334

**Published:** 2026-05-13

**Authors:** Hong Xiang, Na Ren, Ting Wang, Xueyan Zheng, Ziyu Kang, Yanjin Dong

**Affiliations:** ^1^ Institute of Pharmacy (Institute of TCM Health Industrial Technology), Shandong University of Traditional Chinese Medicine, Jinan, China, sdutcm.edu.cn; ^2^ Pharmacy Department, Jinan Municipal People’s Government Organs Outpatient Department, Jinan, China; ^3^ Department of Pharmacy, Jinan Second People’s Hospital, Jinan, China, sdjneye.com; ^4^ Department of Laboratory Medicine, Jinan Second People’s Hospital, Jinan, China, sdjneye.com

**Keywords:** breast cancer, cancer-associated fibroblasts, complement factor B, Mendelian randomization, multiomics integration, PARP inhibitors

## Abstract

**Background:**

Complement factor B (CFB), a key component of the alternative complement pathway, has been implicated in tumor‐related inflammation; however, its causal role and therapeutic relevance in breast cancer remain unclear. By integrating genetic epidemiology, multiomics transcriptomics analyses, and AI‐assisted drug prediction, we aimed to clarify the biological and clinical significance of CFB in breast cancer.

**Results:**

Mendelian randomization analysis suggested a potential causal association between circulating CFB levels and breast cancer risk (OR = 1.048, 95% CI: 1.018–1.078, *p* = 0.001). Single‐cell transcriptomic analysis identified cancer‐associated fibroblasts (CAFs) as the primary cellular source of CFB within the tumor microenvironment. High CFB expression was significantly associated with advanced clinical stage, lymph node metastasis, and poor overall survival and was confirmed as an independent prognostic factor (HR = 2.35, 95% CI: 1.55–3.56, *p* < 0.001). Tumors with elevated CFB displayed immunosuppressive characteristics, including increased regulatory T cells and M2 macrophages, higher TIDE scores, and strong correlations with immune checkpoint molecules. AI‐based drug sensitivity prediction and validation analyses revealed that CFB‐high tumors exhibited enhanced sensitivity to PARP inhibitors. Additional mutation‐related bioinformatic analyses showed that CFB‐mutated tumors were associated with higher TMB, increased MSI‐H proportion, an immunosuppressive microenvironment, and enhanced predicted sensitivity to PARP inhibitors. Mechanistically, CFB expression strongly correlated with PARP1 (*R* = 0.77), and CAF‐derived CFB enhanced olaparib‐induced DNA damage in TNBC models.

**Conclusion:**

Through integrative traditional and AI‐driven approaches, this study supports CFB as a genetically associated risk protein with potential causal relevance and as a prognostic and therapeutic biomarker at both the expression and mutation levels in breast cancer. These findings provide a mechanistic and translational basis for precision therapy targeting CFB‐overexpressing tumors.

## 1. Introduction

Breast cancer is the most commonly diagnosed malignancy among women worldwide. In 2022, approximately 2.3 million new cases were reported, accounting for 11.6% of all cancer cases [1]. Despite substantial advances in early detection and systemic therapy, breast cancer remains a leading cause of cancer‐related death in women [2]. Breast cancer is a highly heterogeneous disease and can be classified into several molecular subtypes, including Luminal A, Luminal B, HER2‐enriched, and basal‐like subtypes, each with distinct biological behavior, therapeutic response, and prognosis [3]. Among these, triple‐negative breast cancer (TNBC) is particularly difficult to treat because of the lack of effective targeted therapies. Therefore, a better understanding of its pathogenesis is urgently needed to support the development of novel therapeutic strategies [4].

The tumor microenvironment (TME) plays a critical role in breast cancer initiation and progression. It is composed of cancer cells, immune cells, fibroblasts, endothelial cells, and extracellular matrix components, which collectively influence tumor growth, invasion, and metastasis [5]. In recent years, immune checkpoint inhibitors have shown therapeutic benefit in several solid tumors. However, the response rate to immunotherapy remains relatively low in breast cancer, and only a subset of patients with TNBC achieve durable responses to PD‐1/PD‐L1 inhibitors [6]. Therefore, a deeper understanding of the immune landscape of the breast cancer microenvironment and the identification of biomarkers predictive of immunotherapy response are of substantial importance for personalized treatment. The complement system is a central component of innate immunity and has traditionally been regarded as a defense mechanism involved in pathogen clearance and inflammation [7]. Increasing evidence indicates that complement signaling also plays complex and context‐dependent roles in the TME. On the one hand, complement activation may restrain tumor growth by supporting antitumor immunity; on the other hand, tumor cells can exploit complement signaling to promote immune escape, angiogenesis, and metastasis [8]. Complement factor B^1^ (CFB), a key serine protease of the alternative complement pathway, binds to C3b and is cleaved by complement factor D to generate the catalytically active C3 convertase (C3bBb), thereby amplifying complement activation [9]. Recent studies have shown that CFB is aberrantly expressed in several malignancies, including hepatocellular carcinoma, pancreatic cancer, and colorectal cancer, and is associated with tumor progression and patient prognosis [10–12]. However, the expression pattern, biological function, and immunological relevance of CFB in breast cancer remain to be fully clarified. Mendelian randomization (MR) uses genetic variants as instrumental variables to infer the causal effects of exposures on disease outcomes, thereby reducing bias from confounding and reverse causality in observational studies [13]. In parallel, single‐cell RNA sequencing enables high‐resolution characterization of the cellular composition and functional states of the TME [14]. In this study, we first applied MR to investigate whether circulating CFB levels are causally associated with breast cancer risk. We then integrated single‐cell transcriptomics and large‐scale cohort data to systematically characterize the expression pattern, prognostic significance, and immune microenvironmental associations of CFB in breast cancer. Finally, we performed drug sensitivity prediction and molecular docking analyses to identify potential therapeutic agents, thereby providing theoretical support for risk assessment, prognostic stratification, and precision treatment in breast cancer.

## 2. Materials and Methods

### 2.1. MR Analysis

A two‐sample MR analysis was conducted to investigate the causal effect of plasma proteins on breast cancer risk. We take the protein quantitative trait locus (QTL) data from the deCODE genetics study and use SOMAscan on 35,559 Icelanders to measure circulating proteins. Pooled breast cancer GWAS data came from the Breast Cancer Association Consortium meta‐analysis (122,977 cases and 105,974 European ancestry controls) through IEU OpenGWAS.

cis‐pQTLs were screened based on the following criteria: SNPs located within ±1 Mb of the transcription start site, genome‐wide significance (*p* < 5 × 10_−8_) and linkage disequilibrium pruning (*r* ^2^ < 0.001, window size 10,000 kb) (Table S1). Instrument strength is measured by the *F*‐statistic, and *F* > 10 indicates that it is good. Causal effect estimation mainly uses inverse variance weighting. Sensitivity analyses include MR‐Egger regression, weighted median method, and weighted mode method [15]. Analysis was performed using the TwoSampleMR R package, with multiple testing correction applied using the Bonferroni threshold (*p* < 1.02 × 10^−5^).

### 2.2. Single‐Cell Transcriptome Analysis

Single‐cell RNA sequencing data (GSE176078) comprise tumor tissues from 26 patients with breast cancer, including 10 patients with TNBC [14], processed using Seurat v4.3.0 [16]. Quality control removed low‐quality cells with fewer than 200 or more than 6000 genes detected per cell, as well as those with mitochondrial gene proportions exceeding 15%. Detailed parameters are provided in Table S1. After data normalization via LogNormalize (scaling factor 10,000), the Top 2000 highly variable genes were selected for principal component analysis. Clustering was performed using the Louvain algorithm (resolution 0.5, Top 30 principal components), with results visualized via UMAP [16]. Cell type annotation was based on classical marker genes (Table S2) and validated using SingleR cross‐validation. Fibroblasts were further stratified into CFB‐high and CFB‐low groups according to the median normalized CFB expression within the fibroblast cluster. Differential expression analysis was performed using the Wilcoxon signed‐rank test, with a significance threshold of adjusted *p* < 0.05 and |log^2^ *F* *C*| > 0.25. Pathway activity scores were calculated using the AddModuleScore function, with gene sets sourced from the MSigDB database [17]. Cell–cell communication analysis was conducted using CellChat [18].

### 2.3. Clinical Cohort Transcriptome Analysis

TCGA–breast invasive carcinoma (BRCA) gene expression data (HTSeq‐FPKM) were downloaded from UCSC Xena, encompassing 1091 primary tumors and 113 adjacent normal tissues. FPKM values were converted to TPM and then log2‐transformed (Pseudocount 1). For survival analysis, patients were stratified into high‐expression and low‐expression groups based on median CFB expression. Clinicopathological association analysis was performed in a subset of 300 TCGA‐BRCA patients with complete clinical annotation and survival information. Differential expression analysis was performed using the limma‐voom workflow [19] with a significance threshold of |log^2^ *F* *C*| > 1.0 and FDR < 0.05. Gene set enrichment analysis was conducted using clusterProfiler in combination with the MSigDB Hallmark gene sets [20]. Significantly, enriched pathways required |*N* *E* *S*| > 1.5, nominal *p* < 0.05, and FDR < 0.25.

### 2.4. Analysis of TME Characteristics

The composition of tumor‐infiltrating immune cells was estimated using the CIBERSORT algorithm combined with the LM22 feature matrix, with 1000 permutation tests performed and only samples with *p* < 0.05 retained [21]. Stromal and immune scores were calculated using the ESTIMATE algorithm [22]. Immune escape potential was quantified using the TIDE score, with immune phenotype scores obtained from the Cancer Immune Atlas database [22]. Correlations between CFB expression and six immune checkpoint molecules (PD‐L1, PD‐1, CTLA‐4, TIGIT, LAG‐3, and TIM‐3) were assessed via the Spearman correlation analysis.

### 2.5. External Validation and Prognostic Model Development

The METABRIC cohort (*n* = 1904) was used for external validation [23]. Expression data (Illumina HT‐12 v3) were normalized using RMA and log2‐transformed. Patients were grouped based on median CFB expression, consistent with the discovery cohort.

Variables with *p* < 0.10 in univariate Cox regression were entered into the multivariable Cox model. Independent prognostic factors were selected using backward stepwise regression based on the Akaike information criterion (AIC). The proportional hazards assumption was evaluated using Schoenfeld residuals. A prognostic nomogram integrating CFB expression, age, and clinical stage was constructed using the rms package. Model discrimination was assessed using time‐dependent ROC curves at 3 years, internal validation was performed using 1000 bootstrap resamples, and clinical utility was evaluated by decision curve analysis.

### 2.6. Pan‐Cancer Analysis

Pan‐cancer analysis was performed across 25 TCGA cancer types, with GTEx normal tissue data incorporated as controls where necessary. Expression data were normalized to TPM, and batch effects were corrected using the ComBat algorithm. Differential expression between tumor and normal tissues was assessed using the Wilcoxon rank‐sum test, with Bonferroni‐adjusted *p* values. Survival analysis was performed using univariate Cox regression to evaluate the association between CFB expression and overall survival. Cancer types with sufficient sample size were further included in immune checkpoint correlation analyses.

### 2.7. Mutation‐Related Bioinformatic Analysis

Somatic mutation data in Mutation Annotation Format (MAF) for TCGA‐BRCA (*n* = 1084) were downloaded from the GDC Data Portal and processed based on the TCGA MC3 pipeline. CFB mutation types were classified according to Sequence Ontology criteria. Tumor mutational burden (TMB) was defined as the total number of nonsynonymous somatic mutations per megabase of coding sequence. Microsatellite instability (MSI) status was obtained from TCGA MSIsensor‐derived results. Molecular subtype annotations were retrieved from the TCGA PanCancer Atlas dataset. Comutation analysis was performed using Fisher’s exact test with Bonferroni correction. For immune characterization, tumor‐infiltrating immune cell fractions were estimated using CIBERSORT with the LM22 signature matrix and 1000 permutations, retaining samples with *p* < 0.05. TIDE and IPS scores were used to evaluate immune escape potential and predicted immunotherapy response. Drug sensitivity was estimated using the GDSC2‐based oncoPredict framework. Functional pathway differences between CFB‐mutant and wild‐type tumors were assessed by GSEA using Hallmark gene sets. In addition, pan‐cancer mutation frequencies of CFB were summarized across selected TCGA tumor types.

### 2.8. Drug Sensitivity Prediction and Molecular Docking

GDSC2 pharmacogenomic data were obtained from the Genomics of Drug Sensitivity in Cancer database [24]. Drug response prediction for TCGA‐BRCA samples was performed using the oncoPredict R package [25], which implements ridge regression models trained on gene expression and drug sensitivity profiles from cancer cell lines. In the present study, the preconfigured GDSC2‐based training framework provided by oncoPredict was used, and TCGA‐BRCA samples were treated as an independent prediction cohort. Model fitting and parameter optimization were performed using the package’s internal cross‐validation procedure, as described in the original publication [24]. Predicted drug sensitivity was represented by estimated IC50 values. The Spearman correlation analysis was then used to assess the association between CFB expression and predicted IC50 values across breast cancer samples. Connectome analyses were carried out to find out candidate compounds that can reverse the CFB‐associated transcriptional patterns [26]. Compounds with negative enrichment scores and significant *p* values were selected for molecular docking validation.

Molecular docking used AutoDock Vina. The CFB protein crystal structure (PDB: 2OK5, resolution 1.9 Å) was used as the receptor after being processed to remove water molecules, add polar hydrogen bonds, and calculate Gasteiger charges. Ligand structures came from PubChem and then did energy minimization. Detailed docking parameters and binding affinity criteria are presented in Table S3. Binding conformation analysis was performed using PyMOL.

To improve comparability between the training and target datasets, expression matrices were processed according to the standard oncoPredict workflow before prediction.

### 2.9. Statistical Analysis

Statistical analysis was done using R v4.2.2. For continuous variables, either parametric or nonparametric tests were used depending upon whether there was normality (Shapiro–Wilk) and homogeneity of variance (Levene’s) tests. Categorical variables were compared by the chi‐square test or Fisher’s exact test. The criteria for choosing specific methods are listed in detail in Table S4. Survival analysis was done using the Kaplan–Meier combined with the log‐rank test, and Cox proportional hazards was used for univariate and multivariate analysis.

All statistical tests were two‐tailed with *α* = 0.05. Exploratory analyses employed the Benjamini–Hochberg method to control the false discovery rate, while confirmatory analyses used the Bonferroni method to control the family‐wise error rate. Correlation strength was determined based on the absolute value of the correlation coefficient: 0.00–0.19 was extremely weak, 0.20–0.39 was weak, 0.40–0.59 was moderate, 0.60–0.79 was strong, and 0.80–1.00 was extremely strong.

## 3. Results

### 3.1. MR Analysis Identifies CFB as a Causally Associated Protein for Breast Cancer Risk

**Figure 1:**
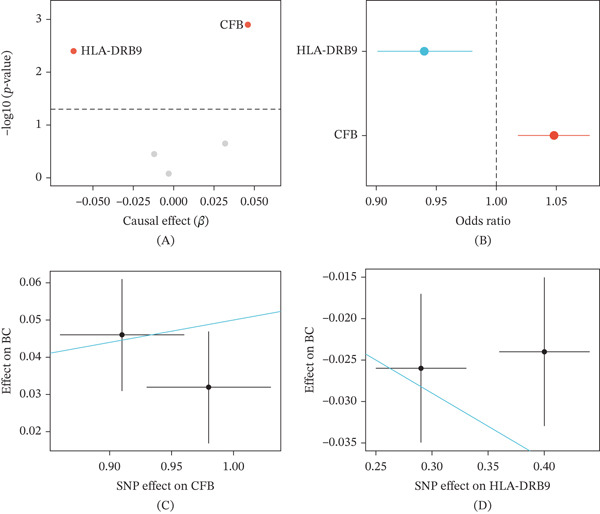
Mendelian randomization identifies circulating CFB as a causally associated protein for breast cancer risk. (A) Volcano plot displaying the causal effect size (beta) and statistical significance (−log10P) of 4907 circulating proteins on breast cancer risk based on two‐sample Mendelian randomization analysis. The horizontal dashed line indicates the Bonferroni‐corrected significance threshold (*p* = 1.02 × 10^−5^). CFB (positive effect) and HLA‐DRB9 (negative effect) reached genome‐wide significance. (B) Forest plot presenting the inverse‐variance weighted (IVW) odds ratios (ORs) with 95% confidence intervals. CFB showed a risk‐enhancing effect (OR = 1.048, 95% CI: 1.018–1.078), whereas HLA‐DRB9 showed a protective association (OR = 0.940, 95% CI: 0.901–0.980). (C, D) Scatter plots illustrating SNP‐exposure versus SNP‐outcome associations. Each point represents an instrumental SNP. The slope corresponds to the IVW causal estimate. CFB genetic instruments (rs4151659 and rs4151667) demonstrated concordant directionality, supporting a robust causal inference. Detailed SNP statistics are shown in the table below the figure.

A two‐sample MR analysis incorporating 4907 circulating proteins is aimed at identifying plasma proteins causally associated with breast cancer susceptibility. After Bonferroni correction, two candidate proteins showed significant causal associations with breast cancer risk (Figure 1A). CFB exhibited a potential causal effect on breast cancer risk (OR = 1.048, 95% CI: 1.018–1.078, *p* = 0.001), while HLA‐DRB9 exhibited a protective association (OR = 0.940, 95% CI: 0.901–0.980, *p* = 0.004) (Figure 1B and Table 1).

**Table 1:** Mendelian randomization estimates for the causal effect of plasma proteins on breast cancer risk.

Exposure	Outcome	Method	nSNP	Beta (SE)	OR (95% CI)	*p* value
CFB	Breast cancer	IVW	2	0.047 (0.015)	1.048 (1.018–1.078)	0.001
HLA‐DRB9	Breast cancer	IVW	2	−0.062 (0.022)	0.940 (0.901–0.980)	0.004

Scatter plots of SNP‐exposure versus SNP‐outcome showed that both CFB and HLA‐DRB9 had the same direction of effect (Figure 1C,D). The two genetic instrumental variables for CFB (rs4151659 and rs4151667) showed the same direction of effect, with exposure effect values of −0.98 and −0.91, respectively, and outcome effect values of −0.032 and −0.046, respectively. No evidence of polygenic effects was found by MR‐Egger regression, thus supporting the validity of causal inference. Given that the complement system plays an important role in the regulation of the TME and the study of CFB is relatively less in breast cancer, subsequent studies focused on in‐depth characterizing the functions of CFB.

### 3.2. Single‐Cell Transcriptomic Analysis Reveals Fibroblasts as the Primary Cellular Source of CFB in Breast Cancer

**Figure 2:**
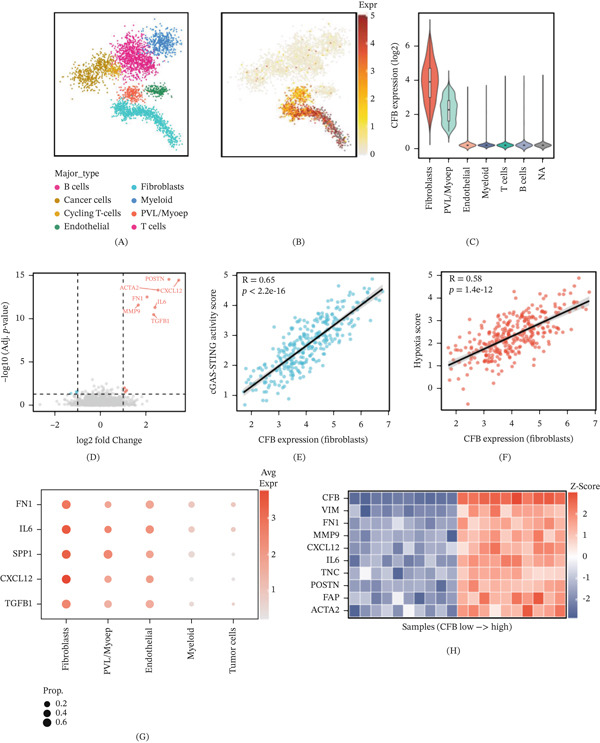
Single‐cell transcriptomic analysis reveals CAFs as the dominant cellular source of CFB and links CFB to protumorigenic pathway activation. (A) UMAP visualization of 26 breast cancer samples (GSE176078), identifying eight major cell populations. (B) UMAP projection of normalized CFB expression across cell clusters, showing enrichment in stromal compartments. (C) Violin plot quantifying CFB expression across cell types; fibroblasts exhibit the highest expression. (D) Volcano plot of differentially expressed genes between CFB‐high and CFB‐low fibroblast subsets (|log^2^ *F* *C*| > 1), adjusted *p* < 0.05). Classical CAF markers (IL6, TGFB1, CXCL12, MMP9, FN1, ACTA2, and POSTN) are significantly upregulated in CFB‐high fibroblasts. (E, F) Positive correlations between fibroblast CFB expression and cGAS–STING activity score (*R* = 0.65) and hypoxia score (*R* = 0.58), respectively. (G) Bubble plot of ligand–receptor‐associated genes across cell types; bubble size indicates expressing cell proportion, and color intensity indicates average expression. (H) Heatmap of CAF‐associated genes stratified by CFB expression status, demonstrating coordinated activation of tumor‐promoting programs.

Eight major cell types were identified by single‐cell RNA sequencing data analysis, including B cells, tumor cells, proliferative T cells, endothelial cells, fibroblasts, myeloid cells, PVL/Myoep cells, and T cells (Figure 2A). CFB expression showed a high degree of diversity across the cellular landscape, and high‐expression clusters were mainly distributed in nonepithelial stromal regions (Figure 2B). From the results of quantitative analysis, it can be seen that the expression level of CFB in fibroblasts is the highest among all the cells detected, followed by PVL/Myoep cells, and the immune cell subsets have the lowest expression (Figure 2C).

Differential expression analysis of the CFB‐high versus CFB‐low subpopulations among the fibroblast population has distinct transcriptional profiles (Figure 2D). The CFB‐high fibroblasts were significantly enriched with the classic tumor‐associated fibroblast (cancer‐associated fibroblast [CAF]) markers, and the most notably upregulated was IL6 (log^2 FC = 2.1, p < 0.001). TGFB1, CXCL12, MMP9, FN1, ACTA2, and POSTN were also coupregulated. CFB expression in fibroblasts positively correlated with cGAS–STING pathway activation scores (*R* = 0.65, *p* < 2.2 × 10^−16^) (Figure 2E) and hypoxia scores (*R* = 0.58, *p* = 1.4 × 10^−12^) (Figure 2F). Ligand–receptor analysis further revealed fibroblasts as the primary source of multiple tumor‐promoting ligands, including TGFB1, CXCL12, SPP1, IL6, and FN1 (Figure 2G). CAF‐associated genes were coordinately upregulated in CFB‐high expression samples (Figure 2H). These findings establish fibroblasts as the primary cellular source of CFB in the breast cancer microenvironment and suggest that CFB expression is closely associated with the activated CAF phenotype.

### 3.3. High CFB Expression Correlates With Adverse Clinical–Pathological Features and Prognosis

**Figure 3:**
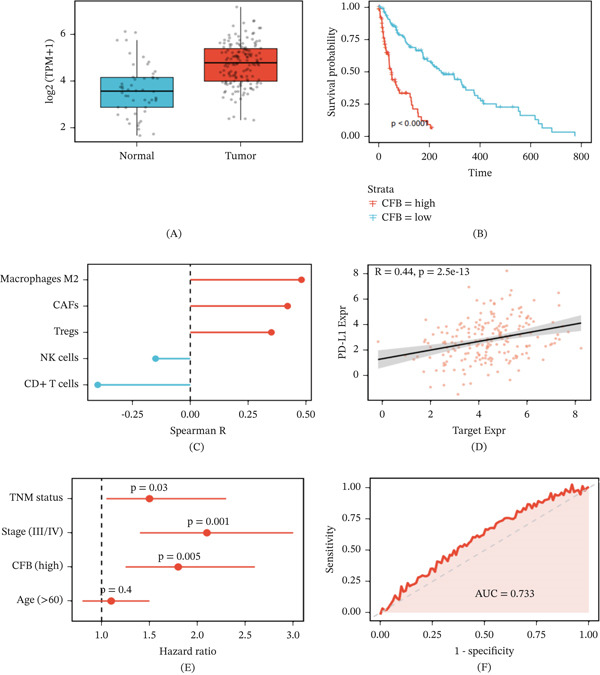
Elevated CFB expression associates with adverse clinicopathological features and independent prognostic significance in breast cancer. (A) Boxplot comparing log2(TPM + 1) CFB expression between TCGA‐BRCA tumor and adjacent normal tissues (two‐sided Wilcoxon rank‐sum test). (B) Kaplan–Meier survival curves stratified by median CFB expression. Log‐rank *p* < 0.0001. (C) Spearman correlation analysis between CFB and tumor‐infiltrating immune cell subsets estimated by CIBERSORT. (D) Positive correlation between CFB and PD‐L1 (CD274) expression with linear regression fit and 95% CI shading. (E) Multivariate Cox proportional hazards model adjusting for age, stage, and TNM classification confirms CFB as an independent prognostic factor (HR = 2.35). (F) ROC curve evaluating diagnostic performance for distinguishing tumor from normal tissue (AUC = 0.733).

CFB expression levels in breast cancer tissue were significantly higher than in adjacent normal tissue (*p* < 0.0001) (Figure 3A). Analysis of clinical and pathological characteristics revealed that high CFB expression was associated with advanced T staging (T3–T4: 48.0% vs. 32.0%, *p* = 0.024), lymph node metastasis (N1–N3: 63.3% vs. 43.3%, *p* = 0.008), advanced clinical stage (III–IV: 56.7% vs. 30.0%, *p* < 0.001), and increased mortality (40.0% vs. 20.0%, *p* = 0.003), while showing no significant association with patient age (Table 2).

**Table 2:** Association between CFB expression and clinicopathological characteristics in breast cancer patients.

Characteristic	Total (*N* = 300)	Low CFB (*N* = 150)	High CFB (*N* = 150)	*p* value
Age (years)				0.412
≤ 60	165 (55.0%)	85 (56.7%)	80 (53.3%)	
> 60	135 (45.0%)	65 (43.3%)	70 (46.7%)	
T stage				**0.024**
T1–T2	180 (60.0%)	102 (68.0%)	78 (52.0%)	
T3–T4	120 (40.0%)	48 (32.0%)	72 (48.0%)	
N stage				**0.008**
N0	140 (46.7%)	85 (56.7%)	55 (36.7%)	
N1–N3	160 (53.3%)	65 (43.3%)	95 (63.3%)	
Clinical stage				**< 0.001**
I–II	170 (56.7%)	105 (70.0%)	65 (43.3%)	
III–IV	130 (43.3%)	45 (30.0%)	85 (56.7%)	
Survival status				**0.003**
Alive	210 (70.0%)	120 (80.0%)	90 (60.0%)	
Dead	90 (30.0%)	30 (20.0%)	60 (40.0%)	

*Note:* Bold values indicate statistical significance (p < 0.05)

The Kaplan–Meier survival analysis revealed significantly poorer overall survival in patients with high CFB expression compared to the low‐expression group (*p* < 0.0001) (Figure 3B). Immune and stromal component analysis demonstrated that CFB expression was positively correlated with immunosuppressive cell populations, including tumor‐associated fibroblasts, M2 macrophages, and regulatory T cells, while negatively correlated with cytotoxic CD8^+^ T cells and natural killer cells (Figure 3C). CFB expression also showed a significant positive correlation with PD‐L1 expression (*R* = 0.44, *p* = 2.5 × 10^−13^) (Figure 3D).

Multivariate Cox regression analysis revealed that after controlling for clinical stage, tumor size, and age, high CFB expression was still an independent poor prognostic factor (HR = 2.35, 95% CI: 1.55–3.56, *p* < 0.001) (Figure 3E and Table 3). Based on the analysis of the receiver operating characteristic curve, it can be seen that the expression of CFB has moderate discriminatory performance to distinguish breast cancer from normal tissue (AUC = 0.733) (Figure 3F).

**Table 3:** Univariate and multivariate Cox proportional hazards analysis of prognostic factors in breast cancer.

Variable	Univariate analysis		Multivariate analysis	
	HR (95% CI)	*p* value	HR (95% CI)	*p* value
Age (> 60 vs. ≤ 60)	1.32 (0.95–1.84)	0.098	1.28 (0.91–1.79)	0.154
Clinical stage (III–IV vs. I–II)	2.45 (1.78–3.38)	**< 0.001**	2.10 (1.45–3.05)	**< 0.001**
Tumor size (T3–T4 vs. T1–T2)	1.85 (1.25–2.74)	**0.002**	1.45 (0.95–2.21)	0.085
CFB expression (high vs. low)	2.85 (1.95–4.16)	**< 0.001**	2.35 (1.55–3.56)	**< 0.001**

*Note:* Bold values indicate statistical significance (p < 0.05)

### 3.4. CFB Expression Correlates With Immune Evasion and Chemotherapy Resistance

**Figure 4:**
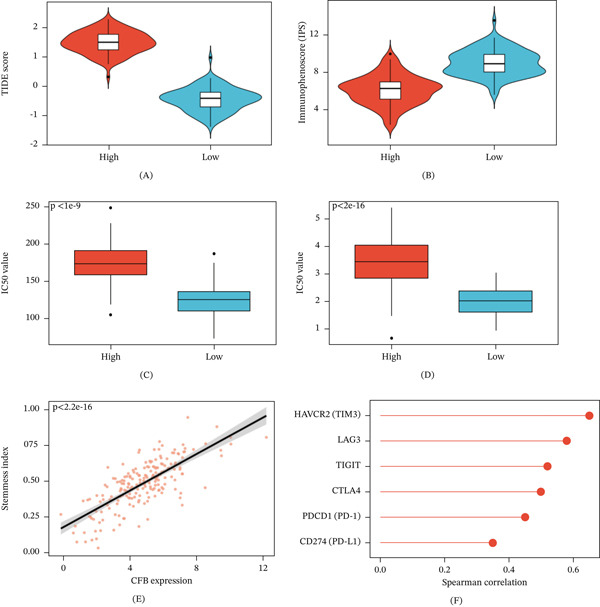
CFB expression correlates with immune evasion, chemotherapy resistance, and tumor stemness in breast cancer. (A) Violin plot comparing TIDE scores between high‐ and low‐CFB expression groups. Tumors with elevated CFB expression exhibited significantly higher TIDE scores (*p* < 0.0001), indicating enhanced immune evasion potential. (B) Violin plot of immunophenoscore (IPS) stratified by CFB expression. CFB‐high tumors displayed significantly lower IPS values (*p* < 0.0001), suggesting reduced predicted responsiveness to anti‐PD‐1/CTLA‐4 immunotherapy. (C) Boxplot of predicted cisplatin IC_50_ values (estimated by the oncoPredict algorithm) according to CFB expression level. Statistical significance was assessed using a two‐sided Wilcoxon rank‐sum test. CFB‐high tumors showed significantly increased IC_50_ values (*p* < 2 × 10^−16^), indicating predicted chemoresistance. (D) Boxplot of predicted paclitaxel IC_50_ values. Statistical significance was assessed using a two‐sided Wilcoxon rank‐sum test. High CFB expression was associated with significantly elevated IC_50_ values (*p* < 2 × 10^−16^), suggesting resistance to taxane‐based chemotherapy. (E) Scatter plot showing the correlation between CFB expression and tumor stemness index (mRNAsi). A strong positive correlation was observed (*R* = 0.73, *p* < 2.2 × 10^−16^), indicating that CFB‐high tumors possess enhanced stem‐like characteristics. (F) Bar plot of Spearman correlation coefficients between CFB expression and inhibitory immune checkpoint molecules, including CD274 (PD‐L1), PDCD1 (PD‐1), CTLA4, TIGIT, LAG3, and HAVCR2 (TIM‐3). All correlations were statistically significant (*p* < 0.0001).

TIDE score analysis showed that patients with high CFB expression had significantly enhanced immune escape potential (*p* < 0.0001) (Figure 4A). According to the IPS analysis, it can be found that the IPS score in the high CFB expression group is significantly reduced (*p* < 0.0001) (Figure 4B), indicating that this group of patients has a weaker response to immune checkpoint blockade therapy.

From the analysis of drug sensitivity prediction results, it was found that in CFB‐high tumors, the predicted IC_50_ values for cisplatin (*p* < 2 × 10^−16^) (Figure 4C) and paclitaxel (*p* < 2 × 10^−16^) (Figure 4D) were predicted to be reduced, suggesting reduced sensitivity to common chemotherapy. The CFB expression was positively correlated with the tumor stemness index (mRNAsi) (*R* = 0.73, *p* < 2.2 × 10^−16^) (Figure 4E), and tumors with high expression of CFB may have stem cell‐like characteristics and thus exhibit resistance to treatment. Furthermore, CFB expression positively correlated with many inhibitory immune checkpoint molecules, PD‐L1 (CD274), PD‐1 (PDCD1), CTLA‐4, TIGIT, LAG‐3, and TIM‐3 (HAVCR2) (Figure 4F).

### 3.5. CFB Expression Regulation Mechanism and External Cohort Validation

**Figure 5:**
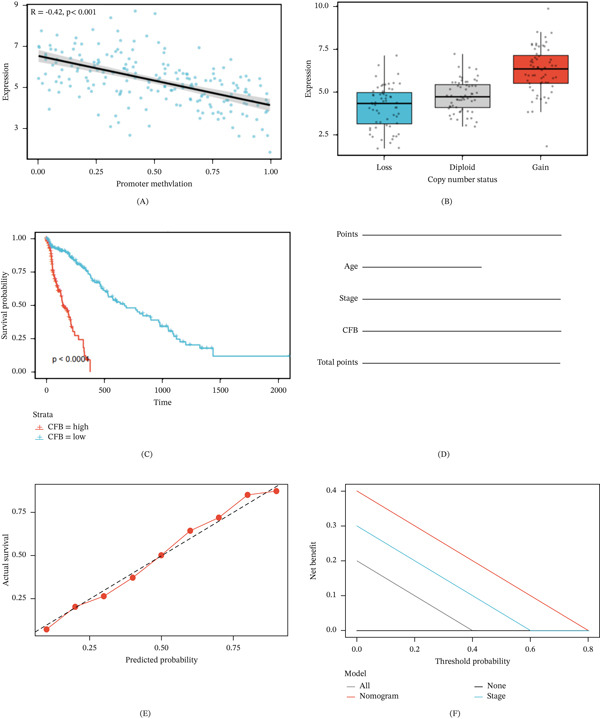
External validation of CFB regulatory mechanisms and prognostic value in breast cancer. (A) Scatter plot showing the inverse correlation between CFB promoter methylation and gene expression (*R* = −0.42, *p* < 0.001). The shaded region represents the 95% confidence interval. (B) Boxplot of CFB expression stratified by copy number variation (CNV) status. Copy number gain (amplification) was associated with significantly increased CFB expression (*p* < 0.001), whereas copy number loss correlated with reduced expression (*p* < 0.01). (C) Kaplan–Meier survival curves of the METABRIC validation cohort (*n* = 1904). Patients with high CFB expression (red) exhibited significantly worse overall survival compared with the low‐expression group (blue) (log‐rank *p* < 0.0001). (D) Prognostic nomogram integrating CFB expression, age, and clinical stage to predict 3‐year overall survival probability. Individual variable scores are summed to generate total points corresponding to predicted survival probability. (E) Calibration curve assessing agreement between predicted and observed 3‐year survival probabilities. The dashed diagonal line represents perfect calibration, and the red curve indicates the performance of the nomogram. (F) Decision curve analysis (DCA) evaluating the clinical utility of the nomogram. Across a range of threshold probabilities, the nomogram (red) provides greater net benefit than the staging model (blue). The gray line represents the treat‐all strategy, and the black line represents the treat‐none strategy.  ^∗^ *p* < 0.05;  ^∗∗^ *p* < 0.01;  ^∗∗∗^ *p* < 0.001.

Epigenetic analysis found a highly negative correlation between the methylation levels of the CFB promoter and gene expression (*R* = −0.42, *p* < 0.001) (Figure 5A), indicating that low promoter methylation promotes CFB transcriptional activation in breast cancer. Copy number variant analysis indicated that the CFB gene amplification samples had significantly higher expression than the diploid samples (*p* < 0.001), and the copy number deletion was negatively correlated with the expression (*p* < 0.01) (Figure 5B).

Gene set enrichment analysis showed that the biological processes were enriched in the CFB‐high expression samples. Epithelial–mesenchymal transition (NES = 2.45), complement activation (NES = 2.38), and inflammatory response (NES = 2.15) were the most highly enriched. The IL6‐JAK‐STAT3 signaling pathway (NES = 1.98), hypoxia (NES = 1.85), and TGF‐*β* signaling pathway (NES = 1.76) were also highly enriched (Table 4).

**Table 4:** Significant gene set enrichment analysis (GSEA) results in CFB‐high breast cancer.

Pathway name	NES	*p* adjust	FDR
HALLMARK_EPITHELIAL_MESENCHYMAL_TRANSITION	2.45	< 0.001	< 0.001
HALLMARK_COMPLEMENT	2.38	< 0.001	< 0.001
HALLMARK_INFLAMMATORY_RESPONSE	2.15	< 0.001	< 0.001
HALLMARK_IL6_JAK_STAT3_SIGNALING	1.98	0.002	0.004
HALLMARK_HYPOXIA	1.85	0.005	0.008
HALLMARK_TGF_BETA_SIGNALING	1.76	0.012	0.018

In the METABRIC validation cohort (*n* = 1904), we also validate that patients with high CFB expression tend to do much worse with regard to overall survival (*p* < 0.0001) (Figure 5C). This agrees with what we found in the discovery set. A prognostic nomogram incorporating CFB expression, clinical stage, and age can forecast the individualized 3‐year survival probability (Figure 5D). Calibration curve analysis demonstrated good agreement between predicted survival probabilities and observed survival rates (Figure 5E). Decision curve analysis confirmed that across a wide range of threshold probabilities, the nomogram incorporating CFB provided superior net clinical benefit compared to models based solely on clinical stage (Figure 5F).

### 3.6. Pan‐Cancer Analysis Reveals the Universal Significance of CFB Across Multiple Malignant Tumors

**Figure 6:**
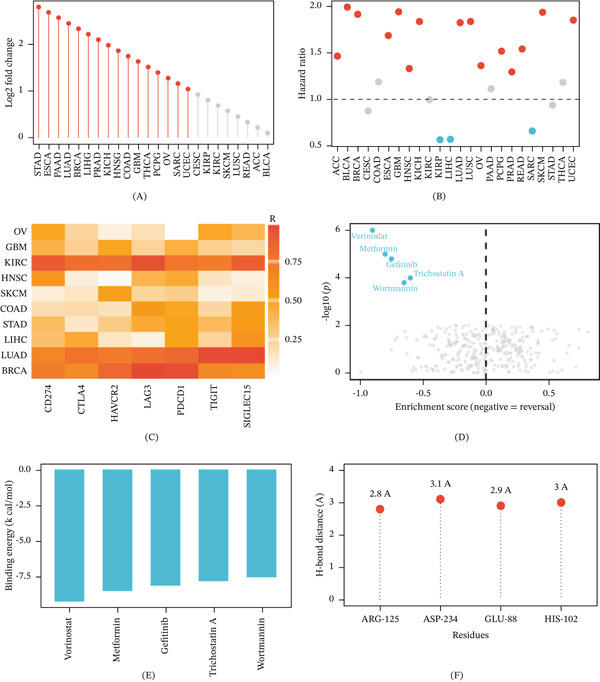
Pan‐cancer expression landscape of CFB and AI‐assisted candidate compound screening. (A) Lollipop plot showing log2 fold change of CFB expression in tumors versus adjacent normal tissues across 25 TCGA cancer types. Red dots indicate significant overexpression; gray dots indicate no significant difference. CFB was notably upregulated in STAD, ESCA, PAAD, LUAD, and BRCA. (B) Lollipop plot displaying hazard ratios from univariate Cox regression across cancer types. Red dots indicate increased mortality risk (HR > 1); blue dots indicate protective association (HR < 1). The dashed line represents HR = 1. Elevated CFB expression was associated with poor prognosis in ACC, KIRC, LUAD, and BRCA. (C) Heatmap illustrating Spearman correlations between CFB and inhibitory immune checkpoint genes across multiple cancer types. Strong positive correlations were observed for CD274 (PD‐L1), CTLA4, HAVCR2 (TIM‐3), LAG3, PDCD1 (PD‐1), TIGIT, and SIGLEC15, particularly in BRCA, LUAD, and KIRC. (D) Connectivity map (CMap) screening plot. The *x*‐axis represents enrichment score (negative values indicate reversal of CFB‐associated transcriptional signatures), and the *y*‐axis shows statistical significance (−log10P). Vorinostat demonstrated the strongest reversal effect, followed by metformin, gefitinib, tigecycline, and oxacillin. (E) Bar chart of molecular docking binding energies between candidate compounds and CFB protein (PDB: 2OK5). More negative binding energy indicates stronger predicted binding affinity. Vorinostat showed the most favorable binding energy (−9.2 kcal/mol). (F) Lollipop plot showing hydrogen bond interactions between vorinostat and CFB residues. Hydrogen bond distances (Å) are indicated. Vorinostat forms stable hydrogen bonds with ARG‐125, ASP‐234, GLU‐88, and HIS‐102.

In the context of a pan‐cancer analysis covering the 25 TCGA cancer types, it could be seen that expression of CFB had been markedly upregulated in tumor tissues over corresponding normal tissues for most of the cancer types. The most upregulation was in gastric adenocarcinoma (STAD), esophageal carcinoma (ESCA), pancreatic adenocarcinoma (PAAD), lung adenocarcinoma (LUAD), and BRCA (Figure 6A). Cox proportional hazards analysis showed that high CFB expression was positively correlated with the risk of death in adrenocortical carcinoma (ACC), clear cell renal cell carcinoma (KIRC), LUAD, and breast cancer but protective in some tumors (Figure 6B). Through the analysis of immune checkpoint‐related 10 types of cancer, we found that CFB expression is positively correlated with inhibitory checkpoint molecules, and in breast cancer, LUAD, and renal clear cell carcinoma, it is especially strongly correlated (Figure 6C).

Connectivity map (CMap) analysis screened compounds which could reverse CFB‐associated transcriptional signatures. Among 1309 compounds, vorinostat had the most reversal effects (*p* < 10^−6^), and metformin, gefitinib, tigecycline, and oxacillin also had some negative enrichment (Figure 6D). Molecular docking simulations revealed that vorinostat exhibited the optimal binding energy with the CFB protein (−9.2 kcal/mol), followed by metformin (−8.5 kcal/mol) and gefitinib (−8.1 kcal/mol) (Figure 6E). Analysis of the binding mode of vorinostat with CFB revealed hydrogen bonds formed with key residues including ARG‐125 (2.8 Å), ASP‐234 (3.1 Å), GLU‐88 (2.9 Å), and HIS‐102 (3.0 Å) (Figure 6F).

### 3.7. Drug Sensitivity Screening Identifies PARP Inhibitors as Potential Therapeutic Agents for CFB‐Overexpressing Breast Cancer

**Figure 7:**
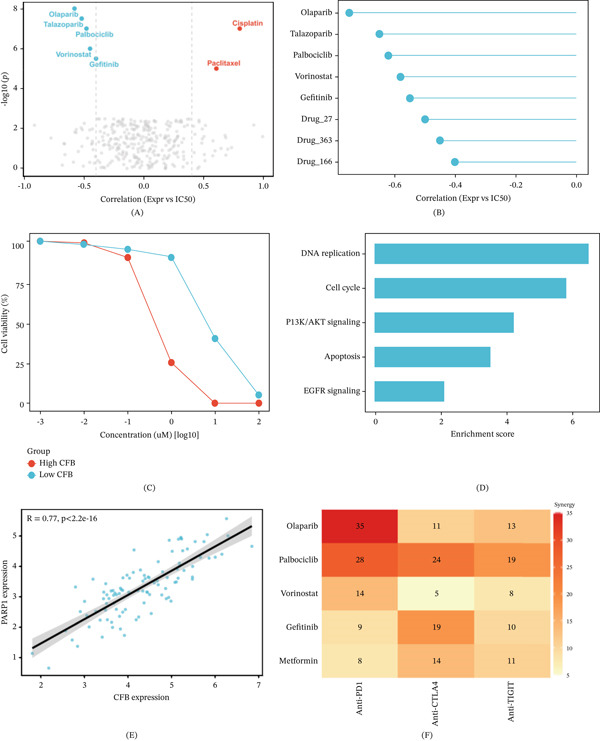
Drug sensitivity screening identifies PARP inhibitors as candidate therapeutic agents for CFB‐high breast cancer. (A) Volcano plot showing correlations between CFB expression and predicted IC_50_ values for 198 compounds from the GDSC database. The *x*‐axis represents Spearman correlation coefficients (negative values indicate increased sensitivity in CFB‐high tumors), and the *y*‐axis represents −log10P. PARP inhibitors (olaparib and talazoparib) and CDK4/6 inhibitor palbociclib were significantly associated with increased sensitivity in CFB‐high tumors. (B) Forest plot ranking candidate compounds based on strength of correlation with CFB expression. Olaparib demonstrated the strongest negative correlation, followed by talazoparib, palbociclib, vorinostat, and gefitinib. (C) Dose–response curves validating differential olaparib sensitivity in breast cancer cell lines stratified by CFB expression. High‐CFB cell lines (red) exhibited lower IC_50_ values compared to low‐CFB cell lines (blue), confirming enhanced PARP inhibitor sensitivity. (D) Pathway enrichment analysis of drug sensitivity‐associated genes in CFB‐high tumors. DNA replication and cell cycle pathways showed the highest enrichment scores, followed by PI3K/AKT signaling, apoptosis, and EGFR signaling pathways. (E) Scatter plot showing a strong positive correlation between CFB and PARP1 expression (*R* = 0.77, *p* < 2.2 × 10^−16^), providing mechanistic support for PARP inhibitor sensitivity in CFB‐high tumors. (F) Heatmap showing predicted synergistic scores for combinations of candidate targeted agents and immune checkpoint inhibitors. Rows represent candidate drugs, and columns represent immune checkpoint inhibitors. Higher values indicate stronger predicted synergy. Olaparib combined with anti‐PD‐1 exhibited the highest predicted synergy score, followed by palbociclib combined with anti‐PD‐1 and anti‐CTLA‐4.

A systematic drug sensitivity screening based on the Genomic Drug Sensitivity Consortium (GDSC) database analyzed the correlation between CFB expression and IC50 values for 198 compounds (Figure 7A). Representative candidate compounds are summarized in Table 5. Tumors with high CFB expression exhibited significantly lower IC50 values for the PARP inhibitor olaparib (*R* = −0.58, *p* < 0.001) and talazoparib (*R* = −0.52, *p* < 0.001), the CDK4/6 inhibitor palbociclib (*R* = −0.48, *p* = 0.002), and the HDAC inhibitor vorinostat (*R* = −0.45, *p* = 0.005) while exhibiting a trend toward resistance to cisplatin (*R* = 0.42, *p* = 0.008) and paclitaxel (*R* = 0.38, *p* = 0.015) (Figure 7B and Table 5).

**Table 5:** Correlation between CFB expression and drug sensitivity (IC50) in breast cancer.

Drug name	Primary target/mechanism	Correlation (*R*)	*p* value	Predicted sensitivity	Docking energy (kcal/mol)
Olaparib	PARP1/2 inhibitor	−0.58	**< 0.001**	High	−8.5
Talazoparib	PARP inhibitor	−0.52	**< 0.001**	High	−8.2
Palbociclib	CDK4/6 inhibitor	−0.48	**0.002**	High	−7.9
Vorinostat	HDAC inhibitor	−0.45	**0.005**	High	−9.2
Cisplatin	DNA crosslinker (chemo)	0.42	**0.008**	Resistant	N/A
Paclitaxel	Microtubule stabilizer	0.38	**0.015**	Resistant	N/A

*Note:* Bold values indicate statistical significance (p < 0.05)

Analysis of drug response data from GDSC cell lines confirmed differential sensitivity to olaparib following CFB expression stratification, with IC50 values significantly lower across all concentrations in CFB‐high cell lines compared to CFB‐low cell lines (Figure 7C). Pathway enrichment analysis revealed that the mechanisms of action for sensitive drugs in CFB‐high tumors converged on DNA replication, cell cycle regulation, PI3K/AKT signaling, apoptosis, and EGFR signaling pathways (Figure 7D). CFB expression showed a strong positive correlation with PARP1 expression (*R* = 0.77, *p* < 2.2 × 10^−16^) (Figure 7E), providing a mechanistic explanation for the observed PARP inhibitor sensitivity.

Predictive analysis for the synergy from combined treatments might generate some drugs that would target along with immune checkpoint inhibitors. The highest predictive synergy score (35) was for olaparib combined with anti‐PD‐1, followed by palbociclib combined with anti‐PD‐1 (28) and palbociclib combined with anti‐CTLA‐4 (24) (Figure 7F). These results indicate that PARP inhibitors may represent a potential therapeutic option for CFB‐high breast cancer, with CFB serving as a candidate biomarker associated with drug sensitivity, and combinations with immune checkpoint inhibition deserve exploration too.

### 3.8. Mutation‐Related Bioinformatic Analysis Reveals Genomic Instability and PARP Inhibitor Vulnerability in CFB‐Altered Breast Cancer

**Figure 8:**
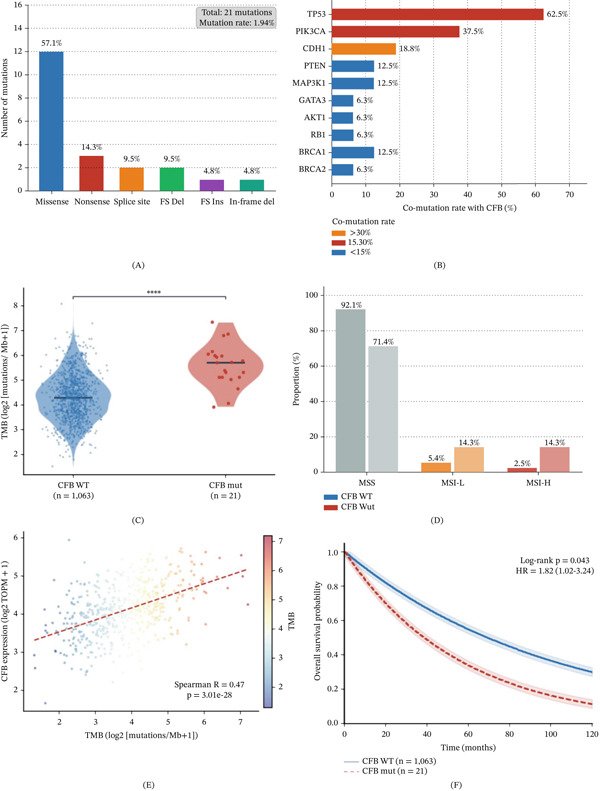
Somatic mutation landscape and genomic instability features of CFB in breast cancer. (A) Distribution of CFB mutation types in the TCGA‐BRCA cohort. Missense mutations constitute the majority, followed by nonsense, splice‐site, frameshift deletions/insertions, and in‐frame deletions. The total number of mutations and overall mutation rate are indicated. (B) Comutation landscape showing the frequency of major gene alterations co‐occurring with CFB mutations. TP53 and PIK3CA exhibit the highest comutation rates. (C) Comparison of tumor mutational burden (TMB) between CFB wild‐type (WT) and mutant (Mut) tumors, presented as log2‐transformed mutations per megabase. Statistical significance was assessed using the Wilcoxon rank‐sum test ( ^∗∗∗∗^ *p* < 0.0001). (D) Distribution of microsatellite instability (MSI) status between CFB WT and Mut groups, including microsatellite stable (MSS), MSI‐low (MSI‐L), and MSI‐high (MSI‐H). Proportions of each category are shown. (E) Correlation between CFB expression (log2 TPM + 1) and TMB. Spearman correlation analysis was performed, and the dashed line represents the linear regression fit. (F) Kaplan–Meier survival curves comparing overall survival between CFB WT and Mut groups. Statistical significance was evaluated using the log‐rank test, and hazard ratio (HR) with 95% confidence interval (CI) is indicated.

To further strengthen the mechanistic interpretation of CFB in breast cancer, we performed a mutation‐related bioinformatic analysis using TCGA‐BRCA somatic mutation data. CFB somatic alterations were identified in 21 of 1084 breast cancer samples, corresponding to a mutation frequency of 1.94% (Figure 8A). Missense mutations were the predominant class (57.1%), followed by nonsense, splice‐site, and frameshift variants (Figure 8A). Comutation analysis showed that CFB‐mutated tumors most frequently harbored concurrent TP53 mutations (62.5%), followed by PIK3CA (37.5%), suggesting that CFB mutation tends to occur in genomically unstable tumor backgrounds (Figure 8B).

CFB‐mutated tumors exhibited significantly higher TMB than CFB wild‐type tumors (median 4.87 vs. 1.42 mut/Mb, *p* < 0.0001) (Figure 8C). In parallel, MSI‐H status was more frequent in the CFB‐mutated group than in the wild‐type group (14.3% vs. 2.5%, *p* = 0.012) (Figure 8D). At the transcriptomic level, CFB expression was positively correlated with TMB (Spearman *R* = 0.47, *p* < 0.001), indicating that CFB dysregulation is closely linked to genomic instability (Figure 8E). Survival analysis further showed that patients harboring CFB mutations had worse overall survival than those with wild‐type CFB (HR = 1.82, 95% CI: 1.02–3.24, *p* = 0.043) (Figure 8F).

**Figure 9:**
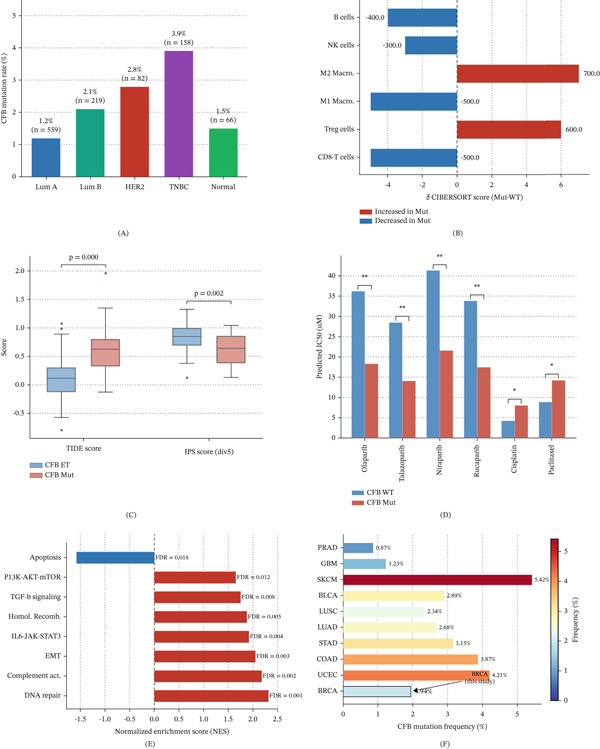
Functional characterization of CFB mutation in breast cancer: subtype distribution, immune landscape, drug sensitivity, and pathway enrichment. (A) Mutation frequency of CFB across different breast cancer molecular subtypes, including Luminal A, Luminal B, HER2‐enriched, and triple‐negative breast cancer (TNBC), as well as normal tissue controls. TNBC exhibits the highest mutation frequency. (B) Differences in tumor‐infiltrating immune cell composition between CFB‐mutant and wild‐type tumors estimated by CIBERSORT. The *x*‐axis represents the difference in infiltration score (Mut − WT). Positive values indicate increased infiltration in the mutant group, while negative values indicate decreased infiltration. (C) Comparison of TIDE scores and immunophenoscore (IPS) between CFB WT and Mut groups. Statistical significance was assessed using the Wilcoxon rank‐sum test, with *p* values indicated. (D) Predicted drug sensitivity (IC50 values) for representative therapeutic agents, including PARP inhibitors (olaparib, talazoparib, niraparib, and rucaparib) and chemotherapeutic drugs (cisplatin and paclitaxel). Lower IC50 values indicate higher drug sensitivity. Statistical comparisons were performed using the Wilcoxon rank‐sum test ( ^∗^ *p* < 0.05 and  ^∗∗^ *p* < 0.01). (E) Gene set enrichment analysis (GSEA) showing significantly enriched pathways in CFB‐mutant tumors. Positive normalized enrichment scores (NESs) indicate pathway activation, whereas negative NES indicates pathway suppression. False discovery rate (FDR) values are shown. (F) Pan‐cancer mutation frequency of CFB across multiple TCGA tumor types. Color intensity represents mutation frequency, highlighting the broader relevance of CFB alterations across malignancies.

Stratified analysis revealed that the mutation frequency of CFB varied across molecular subtypes and was highest in TNBC (3.9%), compared with Luminal A (1.2%), Luminal B (2.1%), and HER2‐enriched tumors (2.8%) (Figure 9A). Immune deconvolution analysis further showed that CFB‐mutated tumors had reduced CD8^+^ T cell and NK cell infiltration, together with increased Treg cell and M2 macrophage infiltration, consistent with a more immunosuppressive TME (Figure 9B). In agreement with these findings, TIDE scores were significantly higher, whereas IPS scores were lower in the CFB‐mutated group, suggesting enhanced immune evasion and reduced predicted responsiveness to immunotherapy (Figure 9C).

Drug sensitivity analysis demonstrated that CFB‐mutated tumors displayed significantly lower predicted IC_50_ values for several PARP inhibitors, including olaparib and talazoparib, whereas sensitivity to cisplatin and paclitaxel tended to be reduced (Figure 9D). GSEA further showed enrichment of DNA repair, homologous recombination, and complement‐related pathways in CFB‐mutated tumors (Figure 9E), providing an additional mechanistic basis for the enhanced PARP inhibitor sensitivity observed in CFB‐altered breast cancer. Pan‐cancer mutation profiling also showed that CFB mutations were detectable across multiple solid tumors, with relatively high frequencies in SKCM, UCEC, COAD, and STAD, supporting the broader relevance of CFB in genomically unstable malignancies (Figure 9F). A comprehensive summary of mutation‐related characteristics, genomic instability features, immune landscape, and drug sensitivity associated with CFB alterations is provided in Table 6.

**Table 6:** Summary of CFB mutation‐associated features in TCGA‐BRCA.

Parameter	CFB wild type (*n* = 1063)	CFB mutated (*n* = 21)	*p* value
Mutation rate (%)	—	1.94%	—
Dominant mutation type	—	Missense (57.1%)	—
TMB (median, mut/Mb)	1.42	4.87	< 0.0001
MSI‐H proportion (%)	2.5%	14.3%	0.012
TIDE score (mean)	0.12 ± 0.35	0.42 ± 0.40	< 0.01
IPS score (mean)	4.2 ± 1.1	3.1 ± 1.2	< 0.05
OS (HR, 95% CI)	Reference	1.82 (1.02–3.24)	0.043
Olaparib IC_50_ (*μ*M)	36.2	18.4	< 0.01
TP53 comutation (%)	30.1%	62.5%	0.003

### 3.9. Characterization and Functional Analysis of CFB‐Expressing CAFs in TNBC

**Figure 10:**
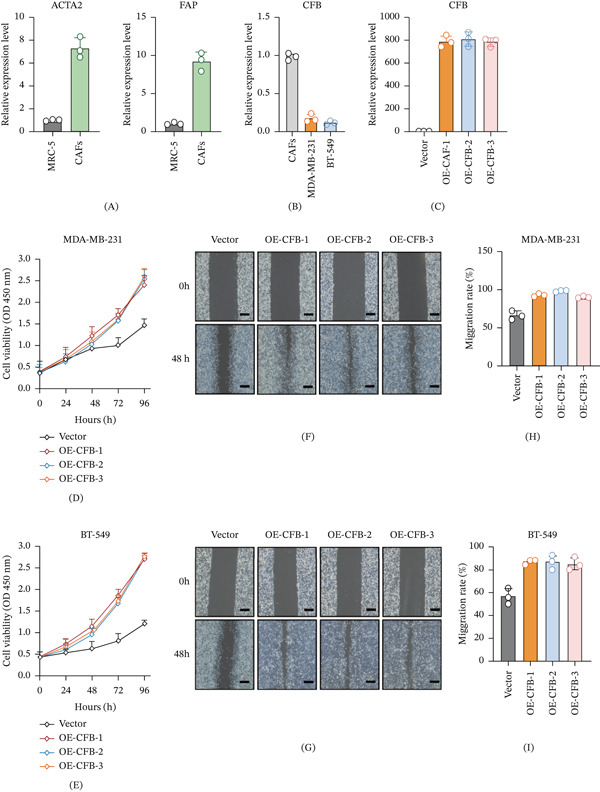
CAF‐derived complement factor B promotes aggressive phenotypes in triple‐negative breast cancer cells.Characterization of CAF phenotype and CFB expression. (A, B) Quantitative real‐time PCR (qPCR) analysis confirming the activated phenotype of commercially obtained breast cancer‐associated fibroblasts (CAFs). mRNA expression of the canonical CAF markers (A) ACTA2 and (B) FAP was significantly upregulated in CAFs compared with normal human fibroblasts (MRC‐5). (C) qPCR analysis showing that CFB mRNA was predominantly expressed in CAFs, with markedly lower expression in TNBC cell lines (MDA‐MB‐231 and BT‐549). (D) Establishment of stable CFB‐overexpressing CAF cell lines. qPCR confirmed robust upregulation of CFB mRNA in three independent clones (OE‐CFB‐1, OE‐CFB‐2, and OE‐CFB‐3) compared with vector‐control CAFs. (E) CCK‐8 assay showing that coculture with CFB‐overexpressing CAFs enhanced the proliferative capacity of MDA‐MB‐231 and BT‐549 cells. (F) Representative images and quantitative analysis of wound‐healing assays showing that coculture with CFB‐overexpressing CAFs accelerated TNBC cell migration. Data are presented as mean ± SD from three independent experiments (*n* = 3).  ^∗^ *p* < 0.05;  ^∗∗^ *p* < 0.01;  ^∗∗∗^ *p* < 0.001. Statistical analyses were performed using one‐way ANOVA followed by Tukey’s multiple‐comparison test, unless otherwise indicated.

To determine the specific cellular origin of CFB, we began by verifying the activated state of our model cells. Commercially obtained breast CAFs exhibited a canonical activated phenotype, with mRNA expression of ACTA2 (*α*‐SMA) and FAP significantly upregulated relative to normal human lung fibroblasts (MRC‐5) (Figure 10A, B). We then compared CFB expression between these validated CAFs and tumor cells. Consistent with single‐cell transcriptomic analysis, CFB mRNA was highly enriched in CAFs, with only minimal expression detected in the TNBC cell lines MDA‐MB‐231 and BT‐549 (Figure 10C). These data conclusively demonstrate that CFB in the TME is predominantly sourced from CAFs.

To functionally dissect the role of CAF‐derived CFB, we performed a series of gain‐of‐function studies. First, stable CAF cell lines overexpressing CFB were established. Commercial breast CAFs were transfected with a CFB overexpression plasmid to generate three independent monoclonal cell lines (designated OE‐CFB‐1, OE‐CFB‐2, and OE‐CFB‐3). Transfection efficiency was confirmed by quantifying CFB mRNA expression levels via quantitative real‐time PCR (qPCR). The results demonstrated a robust and significant upregulation of CFB mRNA in all three OE‐CFB clones compared to control CAFs transfected with an empty vector (Figure 10D), successfully validating the cellular models for subsequent functional assays.

Next, we employed a coculture system to assess the impact of CAF‐derived CFB on the biological behaviors of TNBC cells. Two representative TNBC cell lines, MDA‐MB‐231 and BT‐549, were cocultured separately with the engineered CAFs. The effect on tumor cell growth was first evaluated using a Cell Counting Kit‐8 (CCK‐8) proliferation assay. Monitoring the absorbance at indicated time points (e.g., 0, 24, 48, 72, and 96 h) revealed that coculture with any of the OE‐CFB clone CAFs significantly enhanced the proliferative viability of both MDA‐MB‐231 and BT‐549 cells. This proproliferative effect was particularly evident after 96 h of coculture (Figure 10E).

To further investigate the influence of CAF‐derived CFB on tumor cell migratory capacity, we conducted wound‐healing assays. MDA‐MB‐231 and BT‐549 cells were cocultured with the different CAF groups. Upon reaching confluence, a uniform scratch was created, and wound closure was photographed at 0 and 48 h. Quantitative analysis of the percentage of wound closure area demonstrated that TNBC cells cocultured with OE‐CFB clone CAFs exhibited a significantly accelerated migration rate and a markedly higher wound closure percentage over 48 h compared to those cocultured with vector‐control CAFs (Figure 10F). This finding consistently proves that CFB overexpression in CAFs effectively enhances the in vitro migratory ability of TNBC cells.

### 3.10. CAF‐Derived CFB Differentially Modulates Chemosensitivity in TNBC Cells

**Figure 11:**
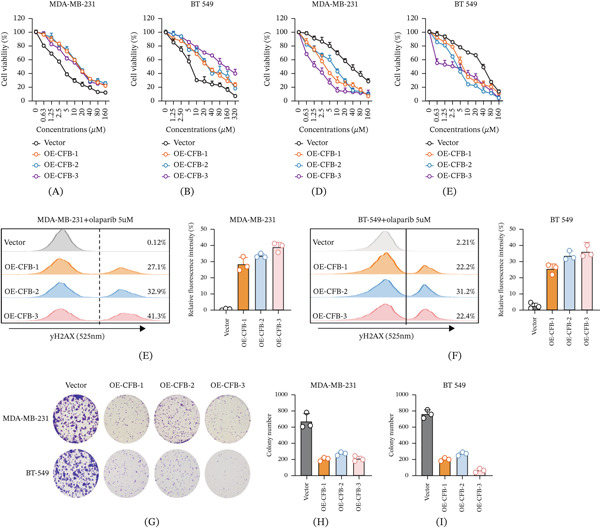
CAF‐derived CFB differentially modulates drug sensitivity in TNBC cells through DNA damage potentiation. CAF‐derived CFB exerts opposing effects on drug responses in TNBC cells. (A, B) Cisplatin dose–response curves in MDA‐MB‐231 and BT‐549 cells. Dose–response curves for cisplatin in (A) MDA‐MB‐231 and (B) BT‐549 cells cocultured with vector‐control or CFB‐overexpressing CAFs (OE‐CFB‐1, OE‐CFB‐2, and OE‐CFB‐3). Cell viability was assessed by CCK‐8 assay. Coculture with CFB‐overexpressing CAFs significantly increased the cisplatin half‐maximal inhibitory concentration (IC50), indicating induced chemoresistance. (C, D) Olaparib dose–response curves in MDA‐MB‐231 and BT‐549 cells.. Dose–response curves for the PARP inhibitor olaparib in (C) MDA‐MB‐231 and (D) BT‐549 cells under the same coculture conditions. CFB overexpression in CAFs substantially reduced the olaparib IC50, denoting enhanced sensitivity. (E, F) Flow cytometric analysis of *γ*H2AX in MDA‐MB‐231 and BT‐549 cells after olaparib treatment. Flow cytometric analysis of the DNA damage marker *γ*H2AX in (E) MDA‐MB‐231 and (F) BT‐549 cells treated with olaparib (5 *μ*M). Representative histograms (left panels) and quantification of *γ*H2AX fluorescence intensity (right panels) show that coculture with CFB‐overexpressing CAFs markedly enhances olaparib‐induced *γ*H2AX accumulation. (G–I) Colony formation assays and quantification in TNBC cells under the indicated coculture conditions. (G) Representative images of colony formation and quantitative analysis of colony numbers for (H) MDA‐MB‐231 and (I) BT‐549 cells treated with olaparib (1 *μ*M). Coculture with CFB‐overexpressing CAFs significantly potentiated the inhibitory effect of olaparib on long‐term clonogenic survival. All data are presented as mean ± SD (*n* = 3 independent experiments).  ^∗^ *p* < 0.05;  ^∗∗^ *p* < 0.01;  ^∗∗∗^ *p* < 0.001 versus vector‐control group. Statistical analyses were performed using one‐way ANOVA followed by Tukey’s multiple‐comparison test, unless otherwise indicated.

The TME is a critical regulator of therapeutic efficacy. To investigate the role of CAF‐derived CFB in modulating drug responses, we performed chemosensitivity assays in a coculture system. Intriguingly, CFB exerted divergent effects depending on the class of therapeutic agent. Coculture with CFB‐overexpressing CAFs conferred significant resistance to the conventional chemotherapeutic cisplatin in both MDA‐MB‐231 and BT‐549 cells. This was evidenced by enhanced cell viability in CCK‐8 assays and a marked increase in the half‐maximal inhibitory concentration (IC50) compared to coculture with vector‐control CAFs (Figure 11A, B). Specifically, the cisplatin IC50 for MDA‐MB‐231 cells cocultured with control CAFs was 3.49 *μ*M, whereas values increased to 17.15, 18.41, and 13.31 *μ*M when cocultured with OE‐CFB‐1, OE‐CFB‐2, and OE‐CFB‐3 CAFs, respectively. A similar, and even more pronounced, effect was observed in BT‐549 cells, where the IC50 rose from 7.15 *μ*M (control) to 33.51, 37.85, and 126.00 *μ*M upon coculture with the respective CFB‐overexpressing CAF clones. These data demonstrate that stromal CFB is a potent inducer of cisplatin resistance in TNBC models.

In stark contrast to its effect on cisplatin, CAF‐derived CFB dramatically enhanced the sensitivity of TNBC cells to the PARP inhibitor olaparib. Dose–response assays revealed that coculture with CFB‐overexpressing CAFs substantially reduced the half‐maximal inhibitory concentration (IC50) of olaparib in both cell lines (Figure 11C, D). Specifically, the olaparib IC50 for MDA‐MB‐231 cells cocultured with control CAFs was 36.17 *μ*M, which plummeted to 4.14, 6.28, and 1.46 *μ*M upon coculture with OE‐CFB‐1, OE‐CFB‐2, and OE‐CFB‐3 CAFs, respectively. A similarly pronounced sensitization was observed in BT‐549 cells, where the IC50 decreased from 30.63 *μ*M (control) to 7.87, 4.28, and 2.10 *μ*M when cocultured with the respective CFB‐overexpressing clones. These marked reductions in IC50 values confirm that stromal CFB potently enhances TNBC cell sensitivity to PARP inhibition.

We next sought to determine the mechanism by which CAF‐derived CFB enhances olaparib sensitivity. To this end, MDA‐MB‐231 and BT‐549 cells were cocultured with either control or CFB‐overexpressing CAFs, treated with olaparib (5 *μ*M), and then subjected to flow cytometric analysis of the DNA damage marker *γ*H2AX. Strikingly, olaparib treatment elicited a significantly stronger *γ*H2AX signal in TNBC cells cocultured with CFB‐overexpressing CAFs versus control CAFs (Figure 11E,F). This result demonstrates that CFB from the tumor stroma exacerbates DNA damage upon PARP inhibition, providing a mechanistic basis for the observed chemosensitization.

We further validated the sustained impact on tumor fitness through long‐term clonogenic survival assays. In these experiments, MDA‐MB‐231 and BT‐549 cells were cocultured with control or CFB‐overexpressing CAFs and treated with olaparib (1 *μ*M). Coculture with CFB‐overexpressing CAFs, in combination with olaparib treatment, led to a significantly greater suppression of colony formation in both TNBC cell lines compared to either condition alone (Figure 11G–I). Collectively, our data demonstrate that CAF‐secreted CFB plays a dual role: It induces resistance to the conventional chemotherapeutic cisplatin, while concurrently sensitizing TNBC cells to PARP inhibition. This chemosensitization is achieved through a mechanism involving exacerbated DNA damage and the significant impairment of long‐term tumor cell survival.

## 4. Discussion

As a central component of innate immunity, the complement system has attracted increasing attention for its roles in tumor initiation and progression [8]. In this study, MR analysis was applied to explore the potential causal relationship between circulating CFB levels and breast cancer risk. Furthermore, by integrating multiomics data, we systematically clarified the expression pattern, prognostic value, immune regulatory role, and potential clinical significance of CFB in breast cancer.

Traditional observational studies are hard to tell between causal and associative, but MR gets around being constrained by confounding and reverse causality with the use of genetics as an instrumental variable [27]. According to large‐scale proteomics data from deCODE Genetics and GWAS data from the Breast Cancer Association Consortium, this study discovered a positive causal link between CFB and breast cancer risk among 4907 circulating proteins. This result is consistent with previous epidemiological reports about the system of complement which causes the chance of breast cancer to become higher [28]. Ajona et al. on lung cancer have illustrated how activating the complement bypass pathway enhances tumor growth through facilitating inflammation and immunosuppression of tumor‐associated [29]. We expand on this by discussing breast cancer and adding genetic causal evidence.

We further investigated the cellular origin of CFB within the TME to better understand its functional role. CAFs are the most abundant stromal cells in the TME. Remake the TME by secreting cytokines, chemokines, and extracellular matrix components in order to promote tumor growth, invasive growth, and metastasis [30]. Single‐cell transcriptome analysis shows that fibroblasts are the main cells of CFB in the breast cancer microenvironment, and CFB‐high fibroblasts also simultaneously express classical CAF markers such as IL6, TGFB1, and CXCL12. Research by Boeker and Kalluri shows that CAFs help tumor cells increase their proliferation and cause them to undergo epithelial‐to‐mesenchymal transition (EMT) via paracrine signaling [31]. Significantly associated with the EMT pathway in this study provides a new molecular insight into this phenomenon. It is especially notable that CFB expression is positively correlated with the activation of the cGAS–STING pathway. The cGAS–STING pathway exerts context‐dependent effects in tumor immunity, contributing to either antitumor responses or immunosuppressive microenvironmental remodeling [32]: It always keeps low‐level activation and causes an immunosuppressed microenvironment instead of antitumor immunity. This could be one of the possible ways for a tumor to do immune escape with high CFB expression.

Poor prognosis of tumors which have a higher level of CFB might be a result of their immunosuppressive microenvironment. Tumors with high CFB expression have a higher infiltration of M2 macrophages and regulatory T cells and a lower infiltration of cytotoxic CD8^+^ T cells and natural killer cells and present typical “cold tumor” features. TIDE scores that are raised and immune phenotype scores that are low also point to the fact that CFB having a high expression is linked to immune escape and resistance to immunotherapy. Research conducted by Shadid et al. shows that the complement activation product C5a recruits myeloid inhibitory cells and suppresses CD8^+^ T cells [33]. Positive correlation between CFB and a few inhibitory checkpoint molecules has also been shown in this paper which is coherent with this immunosuppressive process. These findings suggest that tumors with high CFB expression may benefit from combination strategies involving immune checkpoint inhibitors, whereas monotherapy may be less effective. The molecular mechanism behind the CFB upregulation in breast cancer included multiple layers. DNA methylation is an important epigenetic modification for gene expression regulation; hypomethylation in promoter regions usually results in transcriptional activation [34]. This study found a significant negative correlation between CFB promoter methylation levels and gene expression, indicating that promoter hypomethylation promotes CFB transcriptional activation. At the same time, copy number variation analysis showed that significantly increased copy number samples had higher expression levels, while copy number deletion was negatively correlated with expression. Gene amplification is a feature of the cancer genome and increases its own expression directly [35]. Synergistic action of epigenetic and genetic means might promote the abnormal upregulation of CFB together in breast cancer.

Given the immunosuppressive characteristics and chemotherapy resistance tendency of tumors with high CFB expression, exploring other treatment strategies has clinical significance. In terms of CFB, drug sensitivity tests showed that the tumor overexpression can be more sensitive to PARPi drugs such as olaparib and talazoparib. PARP inhibitors are able to achieve substantial clinical benefit in BRCA‐mutated breast cancers via a synthetic lethality mechanism [36]. Recent research shows that their applications might expand to homologous recombination deficiency–positive but non‐BRCA‐mutated tumors [37]. This study observed that the expression of CFB and PARP1 was positively correlated (*R* = 0.77). PARP1 participates in the DNA damage repair process, and overexpression of PARP1 might be in connection with more genomic disorders and the use of the DNA repair route, which can be seen as a molecular foundation for PARP inhibitors sensitive to CFB overexpression tumors [38]. Combination therapy prediction analysis suggests that there may be a synergistic effect between olaparib and anti‐PD‐1 therapy, which is also in line with the early efficacy reports of recent clinical studies on PARP inhibitors combined with immune checkpoint inhibitors in TNBC [39, 40]. Beyond the PARP inhibitors, we have other connectome hits that could be compounds which might be able to reverse the carcinogenic transcriptional programs driven by CFB. Vorinostat as a histone deacetylase inhibitor is able to restore tumor suppressor gene expression and enhance antitumor immunity by means of epigenetic reprogramming [41, 42]. The most stable docking for the CFB protein (−9.2 kcal/mol) was observed using the docking method. Metformin’s antitumor effects have been verified by different epidemiological studies, preclinical studies, and metformin’s antitumoral mechanisms through activating the AMPK in an mTOR‐suppressing way [43]. These candidate compounds provide new treatment options for CFB‐overexpressing breast cancers, but their direct targeting ability needs to be experimentally confirmed.

To further extend the mechanistic framework beyond expression‐based analyses, we additionally performed mutation‐related bioinformatic analyses in TCGA‐BRCA. Although CFB somatic mutations occurred at a relatively low frequency (1.94%), they were enriched in TNBC and were associated with elevated TMB, increased MSI‐H proportion, and frequent comutation with TP53 and PIK3CA. These findings suggest that CFB alterations may arise preferentially in genomically unstable breast cancers. Notably, CFB‐mutated tumors also displayed a more immunosuppressive microenvironment, characterized by reduced CD8^+^ T cell and NK cell infiltration together with increased Treg and M2 macrophage abundance, as well as higher TIDE and lower IPS scores. These mutation‐level observations are directionally consistent with our expression‐based findings and further support a role for CFB in immune evasion.

Importantly, the mutation‐related analyses also reinforced the therapeutic implication of PARP inhibition. CFB‐mutated tumors showed lower predicted IC_50_ values for olaparib and talazoparib, while GSEA implicated DNA repair and homologous recombination pathways. Together with the strong positive correlation between CFB and PARP1 expression and the in vitro observation that CAF‐derived CFB enhanced olaparib‐induced DNA damage in TNBC cells, these data suggest convergent evidence from both mutation and expression levels supporting PARP inhibitor vulnerability in CFB‐altered breast cancer.

Several limitations should still be acknowledged. In particular, the MR analysis for CFB relied on only two instrumental SNPs, which may limit the robustness of causal inference and warrant cautious interpretation. First, the MR analysis was based primarily on European ancestry datasets, and the generalizability of these findings to other populations requires further validation. Second, the single‐cell dataset mainly included TNBC samples, and the cellular distribution of CFB may differ across luminal and HER2‐positive subtypes. Third, the mutation‐based subgroup was relatively small, which limited statistical power for finer subtype‐level analyses. Finally, drug sensitivity predictions were computational and derived from pharmacogenomic models and therefore require further validation in experimental systems and prospective clinical cohorts. Molecular docking results likewise require biochemical and cellular confirmation.

In summary, this study found that circulating CFB protein levels are associated with breast cancer risk, and the characteristics of CFB include high expression in tumor‐associated fibroblasts, associated with an immunosuppressive microenvironment, and correlated with poor prognosis. As for the possible PARP inhibitors used for CFB‐overexpressing breast cancer patients to explore precision medicine, there will be some new paths. Future work will focus on the functional characterization of the oncogenic machinery and clinical validation of targeted therapeutic strategies of CFB.

## 5. Conclusions

In summary, this study establishes a potential causal association between elevated circulating CFB levels and breast cancer risk through MR, confirming the pivotal role of the complement bypass pathway in tumorigenesis. Integrative multiomics analysis further identifies fibroblasts as the primary cellular source of CFB within the TME, where its overexpression correlates with the infiltration of regulatory T cells and M2 macrophages, fostering an immunosuppressive niche associated with poor prognosis. Notably, while CFB upregulation is linked to predicted reduced sensitivity to conventional chemotherapy, our findings reveal that these tumors exhibit significant sensitivity to PARP inhibitors, a vulnerability mechanistically supported by the strong coexpression of CFB and PARP1. Collectively, evidence from MR, transcriptomic profiling, single‐cell analysis, mutation‐related bioinformatics, and experimental validation supports CFB as an independent prognostic biomarker and a potential therapeutic biomarker associated with PARP inhibitor sensitivity in breast cancer. These results suggest that PARP inhibitors may represent a potential treatment strategy for CFB‐overexpressing breast cancer, with CFB serving as a candidate biomarker associated with therapeutic response, providing a robust theoretical basis for future precision medicine approaches that combine complement targeting with immunotherapy.

## Author Contributions

Hong Xiang contributed to conceptualization, methodology, formal analysis, visualization, and writing–original draft. Na Ren contributed to methodology, data curation, validation, and writing–original draft. Ting Wang was responsible for investigation, resources, and data curation. Xueyan Zheng contributed to software development and validation. Ziyu Kang contributed to conceptualization, supervision, project administration, and writing–review and editing. Yanjin Dong contributed to conceptualization, supervision, project administration, funding acquisition, and writing–review and editing. Hong Xiang and Na Ren are co‐first authors and contributed equally to this work.

## Funding

This study was supported by 2025 Shandong University of Traditional Chinese Medicine Scientific Research Fund Project (Natural Sciences) Grant No. KY2025G03.

## Disclosure

All authors reviewed and approved the final manuscript.

## Ethics Statement

Ethical review and approval were not required for the study on human participants in accordance with the local legislation and institutional requirements. Written informed consent from the patients/participants or patients/participants’ legal guardian/next of kin was not required to participate in this study in accordance with the national legislation and the institutional requirements.

## Conflicts of Interest

The authors declare no conflicts of interest.

## Endnotes

^1^CFB, complement factor B; MR, Mendelian randomization; OR, odds ratio; CI, confidence interval; HR, hazard ratio; OS, overall survival; TME, tumor microenvironment; CAF(s), cancer‐associated fibroblast(s); TAF, tumor‐associated fibroblasts; Treg(s), regulatory T cell(s); TIDE, Tumor Immune Dysfunction and Exclusion; IPS, immunophenoscore; PARPi, poly(ADP‐ribose) polymerase inhibitor(s); IC_50_, half‐maximal inhibitory concentration; GWAS, genome‐wide association study; pQTL, protein quantitative trait locus; SNP, single‐nucleotide polymorphism; LD, linkage disequilibrium; IVW, inverse‐variance weighted; SE, standard error; scRNA‐seq, single‐cell RNA sequencing; UMAP, Uniform Manifold Approximation and Projection; PCA, principal component analysis; DEG(s), differentially expressed gene(s); GSEA, gene set enrichment analysis; NES, normalized enrichment score; FDR, false discovery rate; CIBERSORT, Cell‐type Identification By Estimating Relative Subsets Of RNA Transcripts; ESTIMATE, Estimation of STromal and Immune cells in MAlignant Tumours using Expression data; FPKM, fragments per kilobase of transcript per million mapped reads; TPM, transcripts per million; TCGA, The Cancer Genome Atlas; GEO, Gene Expression Omnibus; GDSC, Genomics of Drug Sensitivity in Cancer; CMap, connectivity map; ROC, receiver operating characteristic; AUC, area under the curve; AIC, Akaike information criterion; DCA, decision curve analysis; CNV, copy number variation; qPCR, quantitative real‐time polymerase chain reaction; CCK‐8, Cell Counting Kit‐8; *γ*H2AX, phosphorylated histone H2AX (Ser139).

## Supporting information


**Supporting Information** Additional supporting information can be found online in the Supporting Information section. Table S1: Detailed information of SNPs used in Mendelian randomization analysis and parameters for single‐cell analysis. Table S2: Marker gene lists for cell type annotation. Table S3: Molecular docking parameters and binding affinity details. Table S4: Criteria for statistical test selection and methodological justification.

## Data Availability

The datasets analyzed in this study are publicly available in TCGA (TCGA‐BRCA), METABRIC, and GEO under accession number GSE176078.
